# The Prader-Willi syndrome Profile: validation of a new measure of behavioral and emotional problems in Prader-Willi syndrome

**DOI:** 10.1186/s13023-024-03045-9

**Published:** 2024-02-23

**Authors:** Elisabeth M. Dykens, Elizabeth Roof, Hailee Hunt-Hawkins

**Affiliations:** Department of Psychology and Human Development, Vanderbilt University, Vanderbilt Kennedy Center, 1 Magnolia Circle, 37203 Nashville, TN USA

**Keywords:** PWS Profile, Behavioral and emotional dysfunction in PWS, Anxiety, Clinical trials, Endpoints

## Abstract

**Background:**

Prader-Willi syndrome (PWS) is a rare, neurodevelopmental disorder caused by the lack of expression of paternally imprinted genes on chromosome 15q11-13. PWS features a complex behavioral phenotype, including hyperphagia, anxiety, compulsivity, rigidity, repetitive speech, temper outbursts, aggressivity, and skin-picking. Questionnaires exist for measuring hyperphagia, but not for the aggregation of other problems that are distinctive to PWS. A PWS-specific tool is needed for phenotypic research, and to help evaluate treatment efficacy in future clinical trials aimed at attenuating PWS’s hyperphagia and related problems. In this 4-phase study, we leveraged our expertise in PWS with feedback from families and specialists to validate the PWS Profile, a novel, informant-based measure of behavioral and emotional problems in this syndrome.

**Results:**

The authors developed a bank of 73 items that tapped both common and less frequent but clinically significant problems in PWS (Phase 1). An iterative feedback process with families and stakeholders was used to ensure content and construct validity (Phase 2). After adding, omitting, or revising items, in Phase 3, we pilot tested the measure in 112 participants. Results were reviewed by an international team of PWS specialists and revised again (Phase 3). The final, 57-item Profile was then administered to 761 participants (Phase 4). Principal component factor analyses (*n* = 873) revealed eight conceptually meaningful factors, accounting for 60.52% of test variance, and were readily interpretated as: Rigidity, Insistence; Aggressive Behaviors; Repetitive Questioning, Speech; Compulsive Behaviors; Depression, Anxiety; Hoarding; Negative Distorted Thinking; and Magical Distorted Thinking. Factors were internally consistent and showed good test-retest reliability and convergent validity with existent measures of behavioral problems. Profile factors were not related to IQ, BMI, or parental SES. Three Profile factors differed across PWS genetic subtypes. Age and gender differences were found in only one Profile factor, Hoarding.

**Conclusions:**

The PWS Profile is a valid, psychometrically-sound questionnaire that already has shown responsivity to treatment in a previous clinical trial. The Profile can extend the reach of future clinical trials by evaluating the impact of novel agents not only on hyperphagia, but also on the emotional and behavioral problems that characterize PWS.

**Supplementary Information:**

The online version contains supplementary material available at 10.1186/s13023-024-03045-9.

## Background

Prader-Willi syndrome is a rare, genetic neurodevelopmental disorder caused by the lack of expression of paternally imprinted genes on chromosome 15q11-q13 [1]. Most cases (~ 70%) are caused by paternal deletions that vary in size (Type I deletions are 0.7 mb larger than Type II deletions). Maternal uniparental disomy (mUPD), or when the child inherits two copies of maternal chromosome 15, is found in approximately 30% of cases. Relatively few individuals have an Imprinting Center Defect, which causes the paternal chromosome 15 to be inactive [2, 3].

PWS features a complex and distinctive behavioral phenotype. Hyperphagia, often cast as the hallmark of PWS, onsets in early childhood and is associated with aberrant hypothalamic functioning and disrupted mechanisms involved in satiety [3]. As a result, hyperphagic individuals with PWS are consistently hungry yet rarely feel sated or full. Given their chronic hunger, they are apt to engage in food-seeking behaviors, sneak food, manipulate others for food, act out in food situations, and deny or lie about their food consumption [4]. Without constant food supervision and external controls, people with PWS risk becoming morbidly obese.

The Hyperphagia Questionnaire was developed to assess these unusual food-related and hunger features of PWS [4]. This 13-item, informant-based measure has also been adapted and used as a primary outcome in several clinical trials aimed at attenuating hyperphagia [5], showing good responsivity to treatment [6, 7].

Beyond hyperphagia, however, people with PWS are prone to other significant behavioral and emotional problems that impede quality of life for them and their families [8]. These include rigid thinking, insistence on sameness, repetitive questioning, compulsivity, anxiety, negative affect, temper outbursts, and skin picking [9–11]. Individuals with paternal deletions are more apt to skin-pick, while those with mUPD are more prone to psychotic episodes and autism spectrum disorder (ASD) [12–14]. PWS is also characterized by mild to moderate deficits in overall cognitive functioning, adaptive behavior, social cognition, and executive functioning [15–17].

Different approaches have been used to describe behavioral and emotional dysfunction in PWS. Some researchers, for example, have determined if people with PWS meet diagnostic criteria for psychiatric disorders [18–20]. But such psychiatric nosology’s as the Diagnostic and Statistical Manual-5-TR [21] and International Classification of Disease-11 [22] are based on the general population, not on those with neurodevelopmental disorders. Recently, DSM 5 diagnostic criteria have been modified for persons with intellectual disabilities [23]. Specifically, the Diagnostic Manual-Intellectual Disability-2 (DM-ID-2), identifies behavioral indicators of psychiatric conditions that are pertinent for individuals with limited cognitive, linguistic, or social functioning [23].

One diagnostic quandary in PWS, repeatedly raised by the FDA and clinical trial sponsors, is the extent to which individuals with PWS meet criteria for anxiety disorders.

Behaviors commonly construed as indices of anxiety in PWS include repetitive questions regarding daily routines, schedules, food, or people, as well as loud, pressured speech, physical agitation, and checking on possessions, schedules, or people [9]. And, in a recent interview study, young adults with PWS consistently expressed feeling anxious, worried, nervous or “stressed out” [24]. A newly validated measure, the PWS Anxiousness and Distress Behaviors Questionnaire, assesses these behavioral indices of anxiety in PWS [25].

Beyond psychiatric diagnoses, researchers have also administered questionnaires to parents or caregivers that tap behavioral and emotional problems, e.g., the Child Behavior Checklist [26] and Strengths and Difficulties Questionnaire [27]. Other researchers have administered behavioral assessments developed specifically for persons with intellectual disabilities, including the Developmental Behaviour Checklist [28] or the Aberrant Behavior Checklist [29]. Similarly, tools developed to diagnose or assess symptoms of Autism Spectrum Disorder (ASD) have been helpful in delineating areas of overlap or discontinuity between PWS and ASD [12, 13].

Questionnaires have several advantages. They are standardized, readily available, convenient to administer and score, and provide profiles of relative strengths and weaknesses. A major disadvantage, however, is that measures normed on other disability groups, or on the general population, do not readily capture the clustering or aggregation of problems that are distinctive to PWS. Looking to other genetic neurodevelopmental disorders, several feature syndrome-specific behavioral assessments or cognitive-linguistic test batteries. Tools have been developed, for example, for individuals with fragile X, Down, Rett and Williams syndromes [30–33]. While some of these are novel tools, others were compiled or adapted from existing assessments.

In PWS as well, a measure is also sorely needed that taps the constellation of behavioral and emotional problems that are particular to this syndrome. A PWS-specific tool can chart phenotypic changes across the lifespan and assess the efficacy of behavioral interventions or pharmaceutical agents in future clinical trials. Indeed, although previous clinical trials in PWS have aimed to attenuate hyperphagia, downstream treatment effects have also been observed in aggression, anxiousness, irritability, and sociability [6, 7, 34].

In this four-phase study, we used an iterative process with stakeholders to develop and validate a new measure, the PWS Profile, designed to capture behavioral and emotional problems in this rare disorder. In doing, so we followed the FDA’s guidelines for Observer-Reported and Patient-Reported Outcomes [35], as well as psychometric principles and statistical analyses involved in questionnaire development. Analyses also assessed relations between the Profile and demographic variables and Profile predictors of anxiety. A single, syndrome-specific index of behavioral and emotional problems in PWS will facilitate future research and complement and extend the Hyperphagia Questionnaire or other outcome measures in future clinical trials.

## Methods and procedures

**Fig. 1:**
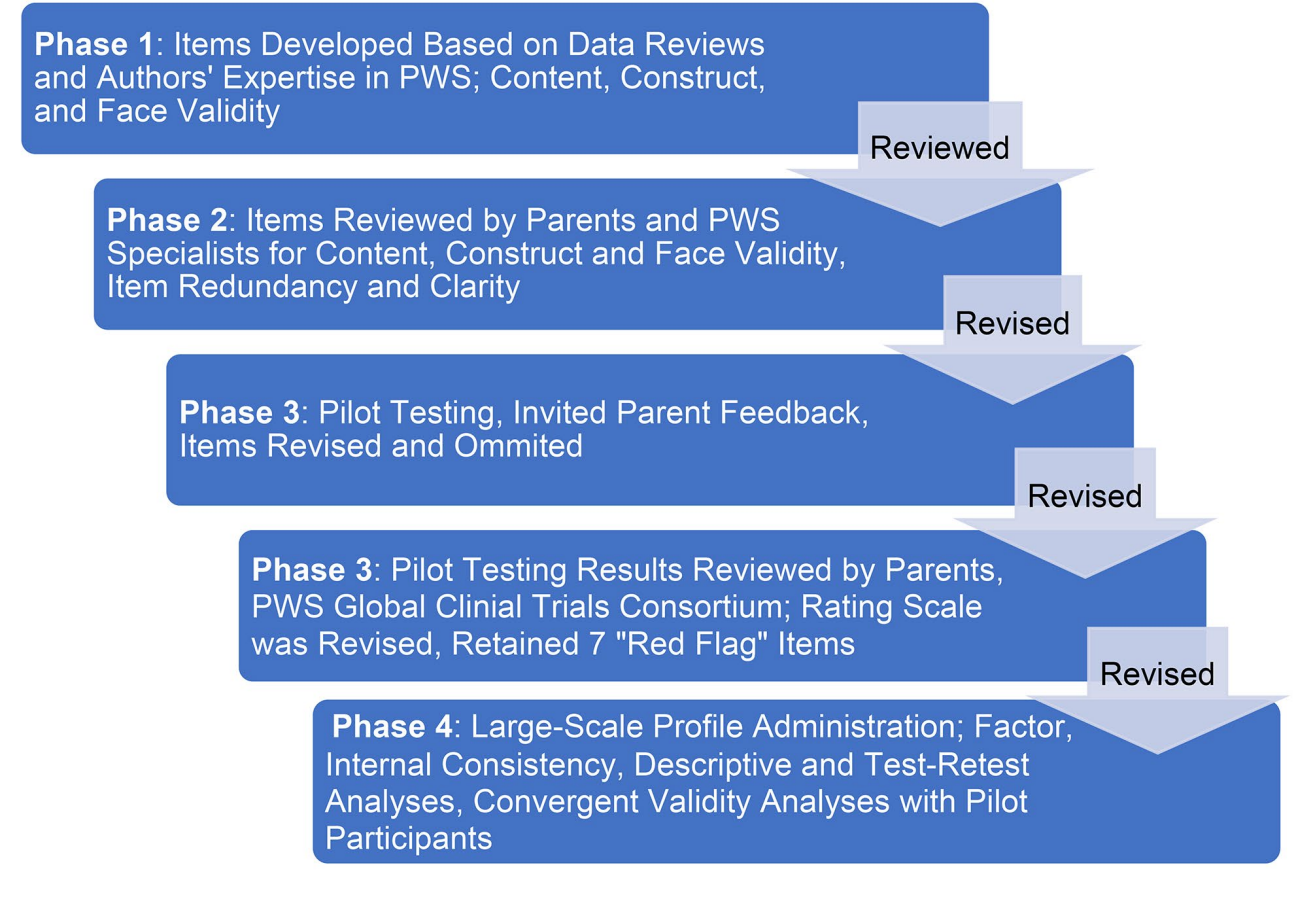
Summary of processes used in developing and validating the PWS Profile

Figure 1 summarizes the four phases of questionnaire development. The Figure also includes how each phase related to subsequent statistical analyses of the Profile’s factor structure, internal consistency, test-retest reliability, and convergent validity.

### Phase 1: profile item development

Consistent with guidelines for validating novel questionnaires [36], and based on our extensive work in PWS, the authors first generated an item-pool. We reviewed data, interviews and clinical consultation notes garnered from 325 families and individuals enrolled in our current and previous PWS research programs. Our goal was to identify frequently observed behaviors, as well as less frequent behaviors that were either very challenging to manage or of high clinical concern. Our team then developed 73 Profile items that included brief descriptions of each behavior. In doing so we aimed to establish both *construct validity* with items that reflected behavioral and emotional problems in PWS, and *content validity* with items that fully represented the major domains of behavioral or emotional dysfunction in PWS.

Items were pertinent for individuals with PWS aged 5 years through adulthood. We established the 5-year-old cut-point as behaviors that characterize PWS typically emerge between 5 and 6 years of age (e.g., needs for sameness, skin-picking, temper tantrums) [37].

### Phase 2: Parental and professional feedback

The pool of 73 items was subsequently vetted by six parents of individuals with PWS. Based on their feedback, a revised version was then reviewed by 10 specialists in PWS, including 2 psychologists, 2 clinical geneticists, 1 school consultant, 1 social worker, 3 PWS group home administrators, and 1 FDA regulatory consultant.

Parents and professionals were asked to provide feedback on the *face validity* of items (e.g., do items on aggression reflect aggression?), clarity of wording and item redundancy. Individuals also assessed *content validity*, specifically if additional items were needed to accurately depict behavioral or emotional problems in PWS [38]. Two additional items were added by the group home administrators (Creates situations to get into the hospital or attention from the police or health professionals; Disrobes when faced with intense emotional state). The revised Profile was then re-examined by the six parents, with an eye toward clarity and understandability of items.

### Informed consent

Prior to Phases 3 and 4 (pilot and large-scale studies), IRB approval was obtained from Vanderbilt University’s IRB Integrated Science Committee. For Phase 3, written, informed consent was obtained from participants using the e-consent function of REDCap (Research Electronic Data Capture), a secure, web-based software platform, designed to support data capture for research studies [39]. After consenting, parents were then invited to complete the PWS Profile on REDCap. Separate IRB approval was obtained for additional data collected from pilot participants. As described below, in Phase 4 additional Profile data were collected via the Foundation for Prader-Willi Research (FPWR) Global PWS Patient Registry. Study approval was also obtained by FPWR’s Internal Review Board. All registrants in the Global PWS Patient Registry gave consent for their de-identified data to be used for research purposes.

### Phase 3: Pilot testing

In the pilot phase, the 75-item Profile was administered to 112 parents of children and adults with PWS (see Participants). Parents were first asked to rate how frequently the behavior occurred over the past six months using a 4-point scale (0 = Almost never, 1 = Sometimes, 2 = Often, 3 = Almost always). For scores > 0, parents were then asked to rate the severity of the problem: 0 = Not a problem; 1 = Mild (annoying but easily redirected); 2 = Moderate (troublesome, needs intervention, causes disruption); 3 = Severe (highly disruptive, very distressing to individual or family). A final open-ended question invited feedback on the Profile.

### Phase 3: Additional pilot data

Four well-established measures were administered to pilot participants to assess convergent validity and correlates of the Profile. Parents completed the Child Behavior Checklist (CBCL) [26], a 113-item measure that assesses Internalizing Problems (consisting of 3 subdomains, Depressed/Anxious, Depressed/Withdrawn and Somaticizing) and Externalizing Problems (consisting of 2 subdomains, Oppositional-Defiant and Aggression). The CBCL also includes additional subdomains: Social, Thought and Attention Problems. Items are rated as (0) not true; (1) somewhat or sometimes true; and (2) very true or often true. Raw scores were used in data analyses.

Parents also completed the Restrictive and Repetitive Behavior Scale-Revised (RBS-R [40], which assesses these behaviors in persons with ASD and other developmental disabilities in 43 items rated on a 4-point scale (0 = behavior does not occur to 3 = behavior occurs and is a severe problem). The RBS-R yields a total score, and scores for 6 subdomains: Stereotyped, Self-Injurious, Compulsive, Ritualistic, Sameness, and Restricted Behaviors.

Parents were administered the Vineland Adaptive Behavior Scales-2 [41], a widely used, semi-structured interview that yields standard scores in three domains of adaptive functioning: Communication, Daily Living Skills, and Socialization, as well as a composite score. Pilot participants with PWS were also individually administered the Kaufman Brief Intelligence Test-2 (KBIT-2) [42], which yields Verbal, Nonverbal and Composite IQ standard scores.

### Phase 3: Preliminary pilot data analyses and additional parental and expert reviews

Preliminary descriptive analyses of pilot data revealed that 18 items were infrequently endorsed or did not correlate well with other items. These 18 items were deleted, resulting in 57 items. The deleted items are listed in Additional File 1.

Descriptive Profile pilot results were then presented to the International PWS Clinical Trials Consortium, a team of expert PWS clinicians, researchers and parents who facilitate clinical trials in this syndrome. Based on their feedback, we simplified the response scale, which some parents in the pilot study found unwieldly. Instead, we adopted a 3-point scale: 1 = Not true or rarely true; 2 = Sometimes true or somewhat true, and 3 = Very true or often true. Doing so facilitated data analyses, reduced administrative time burden for parents, and is consistent with research on optimal scaling [43].

Consortium members also endorsed keeping seven infrequently occurring items in the Profile. These items tapped clinically significant problems in need of further evaluation from mental health professionals (e.g., suicidality, psychosis). These “Red Flag” items were not included in subsequent factor analyses. We did, however, retain them to extend the utility of the Profile as a screener for more severe mental health problems.

### Phase 4: Large-scale profile administration

The final, 57-item version of the Profile was administered to parents of individuals with PWS via The Global PWS Patient Registry, a secure, web-based Registry sponsored by the Foundation for Prader-Willi Research (FPWR) and hosted on the National Organization for Rare Disorders “IAMRARE” registry platform [44]. The goal of the Registry is to identify the natural history of PWS, including medical, developmental, and behavioral features that can inform treatment and future clinical trials [44]. Registrants are asked to consider their offspring’s behavior, social or medical concerns over the last six months, and to complete questionnaires every six months. The six-month time frame was established by FPWR to enhance compliance and reduce parental burden of more frequent assessments. The Registry garnered 761 respondents, and 86% completed the Profile 6 months later.

### Participants

Individuals recruited from Vanderbilt University for the pilot study (*n* = 112) did not differ from participants in the Global Patient Registry (*n* = 761) in age, genetic subtypes, race/ethnicity, parental income or education or Profile scores. As such, analyses combined Profile and demographic data from both sources. Ratings from the Vanderbilt pilot study were recoded to be compatible with the final 3-point scaling used in the large-scale study (i.e., 0 = 1, 1 = 2, 2 & 3 = 3).

Participants thus included a combined total of 873 parents of individuals with PWS (48% males, 52% females) aged 5 to 61 years of age (M = 18.00 years, SD = 10.87). Most respondents (92.1%) were from the U.S. or Canada, 6.7% were from Europe or Australasia, and 9 participants (1.2%) from other regions.

As shown in Table 1, participants were predominately White, resided with their families, and had relatively well-educated parents. Most individuals had paternal deletions (53.7%) or mUPD (33.2%), 3.2% had Imprinting Defects, and 2.0% had translocations. Those with unknown genetic subtypes (8.9%) all received clinical diagnoses of PWS, but parents did not provide genetic testing results. We offset this limitation by ensuring that those with unknown subtypes did differ from their counterparts on any demographic variables or scores on the PWS Profile.

**Table 1:** Demographics of 873 participants with PWS and their families

Demographics	M (SD) or %	
**Age (years)**	18.00 (10.81)	
**Gender**	47.8% M, 52.2% F	
**Body Mass Index**	29.05 (11.27)	
**Race/Ethnicity**:		
White	78.00%	
Multi-Racial, Other	7.40%	
Latino, Hispanic	5.60%	
Asian	4.00%	
Black	2.06%	
Prefer to not answer	2.98%	
**Genetic Subtypes**:		
Deletion	52.7%	
mUPD	33.2%	
Imprinting Defect	3.2%	
Translocation	2.0%	
Unknown	8.9%	
**Living Situation**:		
Parents	84.6%	
Group Home, Residential Care	10.8%	
Supportive Independent Living	4.6%	
**Annual Parental Income**:		
< $29,000	12.1%	
$30,000 to $49,000	13.3%	
$50,000 to $74,000	15.7%	
$75,000 to $99,000	16.6%	
$100,000 to > $200,00	32.1%	
Decline to provide	10.1%	
**Parental Education**:	Mother	Father
High School	10.9%	19.9%
Vocational Training	6.3%	9.6%
Attended College	14.4%	11.5%
Graduated College	44.1%	36.8%
Professional Training	24.3%	22.2%

### Statistical analyses

Factor Analyses of Phase 4 PWS Profile. Exploratory principal component analyses (PCA) were conducted to determine the underlying factor structure of the Profile [45]. Although we considered confirmatory factor analyses, this approach requires a priori designation of items that theoretically tap a latent construct. On face value, some Profile items clearly lend themselves to such theoretical groupings (e.g., items tapping aggressivity). Given the complexities of the PWS phenotype, however, it remained an open question of how most Profile items would cluster together, which is best addressed in PCA [46, 47].

All factor analyses adhered to well-established criteria [45]. These included Kaiser’s criteria with an eigenvalue > 1; visual inspection of the Scree Plot to confirm the number of factors; at least 3 items loading in factors that have a common conceptual meaning; nominal cross-loading across factors; factor loadings and communalities > 0.40; significant Bartlett’s Test of sphericity; and a Kaiser-Meyer-Olkin measure of sampling adequacy that was close to 1.

Separate PCA’s were conducted using orthogonal (i.e., varimax) and oblique rotations (i.e., equimax), allowing us to compare the results of both to determine the most parsimonious, conceptually meaningful solution. These analyses yielded the same number of factors, and similar percentages of variance and factor loadings. As results were similar across rotations, final analyses used the orthogonal solution [46].

Internal Consistency. As the Profile is multidimensional, an overall Cronbach’s alpha was not established for the entire instrument [48]. Instead, alphas were calculated for each factor that represented a common conceptual domain.

Convergent Validity. Pearson correlations were conducted between the total Profile mean score with the CBCL Internalizing and Externalizing Domains, and the total RBS-R score. If significant, we then followed up with correlations between Profile factors and subdomains of the CBCL or RBS-R. To correct for Type II errors, only correlations ≥ 0.40 and with p’s < 0.001 are reported.

Test-Retest Reliability. Most parents in the FPWR Patient Registry, 86%, completed the Profile at Time 1, and again 6 months later. To minimize test-retest measurement error, we ensured that raters were the same across assessments and that individuals with PWS were not enrolled in clinical trials aimed at attenuating their hyperphagia and related behavior problems.

Even so, the 6-month interval is longer than typically used in test-retest analyses. As such, we first computed Intraclass Correlation Coefficients (ICCs), which incorporate both the degrees of agreement and correlations between Time 1 and Time 2 [49]. ICC analyses were based on a single measurement and absolute agreement using a two-way, mixed-effects model in which participant effects were randomized and measure effects were fixed.

Second, to capture individual fluctuations over time, difference scores were calculated between Time 1 and 2 mean scores. We reasoned that on a 3-point scale, a change of +/- 0.5 point represented a noteworthy shift. To check this assumption, we also calculated the mean of each factor’s standard deviation at Time 1 and 2 and identified participants with difference scores that were higher or lower than ½ of each factors mean standard deviation.

Profile Factors and IQ, Adaptive Behavior and Demographics. Age in pilot participants was negatively correlated with KBIT-2 IQ and Vineland Adaptive Behavior scores. As such, partial correlations controlling for age were conducted between Profile factors and KBIT-2 and Vinland scores. Using the combined large data set, t-tests, Chi-Squares, or correlations were conducted to assess relationships between Profile factors and PWS genetic subtypes, age, gender, Body Mass Index (BMI), parental SES, and region of residence.

Predictors of Anxiety. Two regression analyses were conducted in pilot participants to address lingering questions related to anxiety in PWS. In the first regression, the dependent variable was the CBCL Depressed/Anxious subdomain, and predictors included five Profile factors: Depressed, Anxious; and Distorted Negative Thinking, as well as three other factors presumed to be indicative of anxiety in PWS; Rigidity, Insistence; Compulsive Behaviors; and Repetitive Questioning, Speech [9].

Because the CBCL subdomain includes symptoms of both depression and anxiety, we conducted a binary logistic regression to home in on anxiety disorders. The dichotomous dependent variable was the presence or not of Generalized Anxiety Disorder (GAD). These diagnoses were previously obtained in our research program using the DSM 5 version of the KSADS psychiatric interview [50]; 36% of pilot participants received this diagnosis. This logistic regression determined the effects of four Profile factors (Depression, Anxiety; Compulsive Behaviors; Rigidity, Insistence; and Repetitive Questioning, Speech) on the likelihood of having GAD [51].

## Results

### Factor analyses

As summarized in Table 2, the final factor analysis yielded 8 factors and accounted for a total of 60.52% of test variance. Items loading onto each factor shared a common conceptual meaning and factors aptly reflected the multifaceted problems seen in PWS. The labels readily assigned to factors included: Rigidity, Insistence.

**Table 2:** Profile factor labels, eigenvalues, % variances, Cronbach’s alphas, and mean factor scores

PWS Profile Factor Labels	Rotated Eigenvalue	% Variance	Cumulative % Variance	Alphas	Mean (SD)
(1) Rigidity, Insistence	5.309	12.950	12.950	0.87	2.02 (0.51)
(2) Aggressive Behaviors	4.062	9.907	22.856	0.87	1.72 (0.51)
(3) Repetitive Questioning, Speech	3.371	8.222	31.079	0.82	2.24 (0.56)
(4) Compulsive Behaviors	2.593	6.325	37.403	0.74	2.02 (0.57)
(5) Depressed, Anxious	2.444	5.961	43.364	0.72	1.62 (0.46)
(6) Hoarding	2.430	5.927	49.291	0.80	2.00 (0.72)
(7) Negative Distorted Thinking	2.355	5.745	55.035	0.81	1.70 (0.62)
(8) Magical Distorted Thinking	2.248	5.483	60.518	0.72	1.64 (0.59)
Skin-Picking*				0.54	2.12 (0.68)
Total PWS Profile				NA	14.93 (2.06)

*Note. Not included in final factor analyses as this domain only includes 2 items, with a low alpha. They are, however, retained in the Profile as they frequently occur in PWS

(9 items); Aggressive Behaviors (9 items); Repetitive Questioning, Speech (5 items); Compulsive Behaviors (4 items); Depressed, Anxious (7 items); Hoarding (3 items); Negative Distorted Thinking (3 items); and Magical Distorted Thinking (3 items). Table 2 summaries each factor’s rotated eigenvalues and associated percentages of variance. As factors contained different numbers of items, mean scores for each factor are also summarized in Table 2.

The final analysis did not include three items that failed to load onto any factor, and three that were deemed redundant. These 6 items were removed one at a time to determine any effects on remaining items or factors; these 6 items are noted in Additional File 2.

Preliminary analyses also revealed that one factor solution consisted of just 2 items, skin-picking, and nail biting or picking. Both were frequently endorsed (81%% and 57.4%, respectively), with strong factor loadings (0.823 and 0.739, respectively). Even so, this two-item factor had poor internal consistency (Cronbach’s alpha = 0.54) and did not load onto other factors tapping repetitive behavior. Previous work also finds that skin-picking stands alone relative to other repetitive behaviors in PWS [52]. Because skin-picking is prevalent in PWS, these two items were retained in the Profile but not included in the final factor analysis.

Table 3 displays the Profile items that loaded onto each factor, as well as the factor loading and communality of each item. Factor loadings were strong, with 14 items that ranged from 0.40 to 0.59; 15 items from 0.60 to 0.69; and 13 ranged from 0.70 to 0.83 [47]. Similarly, communalities indicated that all items were valuable in contributing to the test variance of their respective factors.

**Table 3:** Items included in Profile Factors, Factor Loadings, Communality’s, and Frequency of Item Endorsement

Factors and Profile Items	Factor Loading	Communality	% Sometimes or Somewhat True	% Very True or Often True
**(1) Rigidity, Insistence**				
Avoids taking responsibility for mistakes	0.681	0.600	42.4%	33.3%
Disagrees for the sake of disagreement	0.672	0.524	37.8%	18.3%
Insists his/her own opinions are correct even when facts contradict them (e.g., You changed your hair color. No, it’s the same color. No, you changed it.)	0.671	0.632	37.5%	29.4%
Difficulty taking others point of view (has difficulty seeing that others have views, needs, or emotions that are different from their own)	0.651	0.627	44.5%	31.3%
Seems to finally understand something only after other people get really mad	0.609	0.430	44.1%	18.0%
Rigid or concrete thinking, things are either black/white, all/none, right/wrong	0.600	0.623	36.8%	44.4%
Difficulty controlling volume of their voice	0.588	0.527	36.9%	38.9%
Wants his/her own way all the time, unwilling to compromise	0.549	0.568	51.4%	24.4%
Needs others to acknowledge that he/she is right or doing things correctly (overly concerned about being wrong, or overly defensive when told he/she is incorrect)	0.434	0.542	36.2%	39.1%
**(2) Aggressive Behaviors**				
Physically aggressive (e.g., swings at others if too close; may throw items, hit, kick, spit)	0.822	0.752	39.1%	8.8%
Has instances of rage (anger directed toward others that may cause injury or harm property)	0.792	0.702	34.3%	9.1%
When angry, will destroy items of personal value	0.760	0.673	30.2%	10.0%
Is verbally aggressive (e.g., argues, yells, screams, loud voice-demands to be heard, gets in your face, may make threatening gestures)	0.650	0.645	41.8%	18.8%
Seeks to intimidate (gets to close when angry, attempts to appear threatening)	0.61	0.590	24.0%	8.0%
Has temper tantrums contained to him/herself (e.g., cries, screams, throws self on ground, holds breath, stomps feet)	0.574	0.500	47.4%	21.2%
Needs to test boundaries or rules, especially with new people or in new settings	0.455	0.542	36.2%	29.2%
Acts impulsively before considering the consequences	0.435	0.574	44.8%	27.8%
Shuts down after aggressive or upsetting episode, doesn’t respond to name or redirection	0.404	0.490	35.8%	24.1%
**(3) Repetitive Questioning, Speech**				
Repeatedly asks about people, events, or situations	0.768	0.743	32.5%	53.3%
Is stuck on specific topic(s) in conversations (starts conversations with topic, brings conversation back to topic)	0.756	0.728	34.6%	53.1%
Asks or says the same or similar things over and over again	0.714	0.689	32.6%	54.3%
Overly preoccupied with a certain person(s), (e.g., talks/ asks about person, needs reassurance about that person, needs to be in contact with person)	0.605	0.566	35.7%	28.0%
Speech is intense, pressurized, or fast, interrupts others to say things	0.507	0.570	38.8%	33.6%
**(4) Compulsive Behaviors**				
Insists that daily routines (e.g., events, meals, self-care, routes to home, school, work) happen in the same way	0.670	0.614	37.1%	43.6%
Arranges items until they are just right (e.g., cards, toys, books, collections)	0.656	0.591	32.0%	23.0%
Difficulty changing from one activity to another, gets stuck	0.632	0.625	48.5%	34.8%
Checks on things (e.g., items in room, backpack) to make sure they are still there and as they left them	0.580	0.568	33.7%	26.6%
**(5) Depressed, Anxious**				
Looks/seems sad or unhappy	0.710	0.648	30.0%	4.7%
Has a negative outlook on life (expects that things won’t go his/her way, pessimistic)	0.614	0.666	21.8%	8.6%
Doesn’t actively seek the company of others (unlikely to join in social activities)	0.581	0.470	38.4%	17.3%
Overly focused on minor bodily issues or health concerns, complains of aches or fatigue	0.547	0.420	38.9%	15.9%
Easily frustrated (may give up quickly or become upset with tasks that require any effort)	0.476	0.527	48.9%	25.3%
Seems uptight or wound up or unable to relax	0.420	0.483	38.4%	13.2%
Becomes physically agitated when nervous (e.g., moves around, paces, rocks, rubs hands together, moves with intensity)	0.410	0.454	31.7%	25.1%
**(6) Hoarding**				
Saves or collects items that you would likely throw away or recycle (e.g., junk mail, old school papers, boxes, wrappers, notes, pictures, old pencils, magazines)	0.830	0.771	25.9%	33.2%
Has difficulty throwing or giving away items that he/she doesn’t use (needs to keep items as they are mine or just in case items are needed)	0.824	0.802	29.5%	35.4%
Saves or collects items around a specific interest or theme (e.g., baby dolls, puzzles, Harry Potter, trains, animals, Disney characters, TV shows)	0.710	0.611	28.2%	40.4%
**(7) Negative Distorted Thinking**				
Misinterprets or over personalizes others’ actions as negative (e.g., a strangers’ frown means they are mad at him/her)	0.710	0.705	32.2%	15.9%
Feels that others intentionally bother or annoy them	0.662	0.657	37.2%	14.6%
Easily slighted, feels as if treated unfairly	0.630	0.672	35.9%	16.9%
**(8) Magical Distorted Thinking**				
Insists that strangers are people they know (e.g., That girl was in my class last year)	0.764	0.705	28.9%	16.4%
Misinterprets others’ actions as overly positive (e.g., a strangers’ smile, or casual greeting means they are a friend)	0.634	0.600	39.3%	19.6%
States that TV or other fictional characters are real (e.g., Harry Potter, Twilight, Disney)	0.602	0.500	22.9%	14.3%

To provide another perspective on Profile items, Table 3 also includes the relative frequencies of items that were rated as “sometimes or somewhat true” and “very true or often true.” Similarly, Table 4 summarizes the relative frequencies of the seven clinically significant Red-Flag items.

**Table 4:** Relative frequencies of seven clinically significant “Red Flag” items

Red Flag Items	% Sometimes or Often
Rectal picking (may not directly observe this behavior but evidence may be on hands, underwear, or sheets).	20.3%
Pulls hair out, including head, eyebrows, lashes*	17.9%
Runs away	13.4%
Makes gestures or behaves in ways that could cause serious self-harm (e.g., runs into street/traffic, opens door in moving car, injures self with intent).	20.6%
Makes statements such as I don’t want to be alive, It would be better if I weren’t here, or I can’t do this anymore.	16.6%
Sees or talks to people who are not there.	26.2%
Reports hearing voices that others do not (could be scary or friendly voices).	9.7%

**Note** * This percentage was derived from the pilot study. Although not included in the large-scale study, we deemed it clinically important to include it as a Red Flag. This decision was also endorsed by parents and PWS specialists involved in Phase 2 of the study

### Internal consistency

Conventional rules of thumb suggest that alphas > 0.70 and < 0.90 are considered good [53]. As noted in Table 2, five Profile factors had alphas > 0.80, and three > 0.70. Importantly, Cronbach’s alphas were also calculated with Time 2 data, and these values were comparable to Time 1 alphas, differing by no more than 0.03.

**Table 5:** Time1 and Time 2 Intra Class Correlations (ICCs) with 95% Confidence Intervals (CI), and percentages of participants with stable, worse, or improved Profile factor scores over time

PWS Factors	ICCs (95% CI)	% Stable	% Worsened	% Improved
Rigid, Insistent	0.78 (0.74-0.81)	87.0%	5.4%	7.6%
Aggressive Behaviors	0.78 (0.74-0.81)	86.8%	7.0%	6.2%
Repetitive Questioning, Speech	0.74 (0.70-0.77)	80.0%	10.7%	9.3%
Compulsive Behaviors	0.70 (0.66-0.73)	64.3%	18.8%	16.9%
Depressed, Anxious	0.65 (0.60-0.69)	70.9%	13.5%	13.6%
Hoarding Behaviors	0.75 (0.72-0.79)	71.0%	14.7%	14.3%
Negative Distorted Thinking	0.68 (0.64-0.72)	71.7%	13.5%	14.8%
Magical Distorted Thinking	0.70 (0.66-0.74)	77.7%	11.0%	11.3%

**Table 6:** Correlations between the CBCL and RBS-R total and subdomains scores with the total Profile and factor scores

Total CBCL, RBS-R Scores	Total Profile Score
CBCL Internalizing	*r* =.51
CBCL Externalizing	*r* =.57
RBS-R	*r* =.52
**CBCL or RBS-R Subdomains**	**Specific PWS Profile Factors**
CBCL Depressed/Anxious	Depressed, Anxious *r* =.56 Distorted Negative Thinking *r* =.46 Rigid, Insistent *r* =.44
CBCL Aggression	Aggressive Behaviors *r* =.68 Rigid, Insistent *r* =.46 Repetitive Questioning, Speech *r* =.41 Compulsive Behaviors *r* =.44 Depressed, Anxiousness *r* =.48
CBCL Social Problems	Rigid, Insistent *r* =.43 Aggressive Behaviors *r* =.46 Depressed, Anxious *r* =.51 Distorted Negative Thinking *r* =.45
RBS-R Sameness	Repetitive Questioning, Speech *r* =.42 Aggressive Behaviors *r* =.47 Compulsive Behaviors 4 = 0.48
RBS-R Ritualistic Behaviors	Repetitive Questioning, Speech *r* =.41 Compulsive Behaviors *r* =.48
RBS-R Compulsive Behaviors	Compulsive Behaviors *r* =.53 Hoarding *r* =.47
RBS-R Stereotypical Behavior	Repetitive Questioning, Speech *r* =.43

**Note** All p’s < 0.001

### Convergent validity

Table 6 presents the significant correlations between the total Profile mean score with CBCL Internalizing and Externalizing Domains, and the total RBS-R score. Given these significant correlations, Table 6 also includes follow-up correlations that were ≥ 0.40 between Profile factors and the subdomains of the CBCL or RRBS-R. Resulting correlations were logical (e.g., between the CBCL Aggression subdomain and Aggressive Behaviors Profile factor) or as discussed below, consistent with the PWS phenotype.

### Test-retest reliability

ICCs and their corresponding 95% confidence intervals are presented in Table 5. Based on conventional criteria, ICCs were all in the moderate to good range [49]. Both approaches to assessing individual variability between Time 1 and 2 resulted in remarkably similar, and at times identical, classifications of participants who had stable, improved, or worse scores. Table 5 summarizes the percentages of participants in these 3 categories based on the more intuitive method of a ½ change in average-factor scores over time; most participants showed stable scores.

### Relations with IQ, adaptive behavior and demographics

IQ and Adaptive Behavior. No significant age-corrected correlations were found between Profile factors and K-BIT-2 Verbal, Nonverbal or Composite IQ scores. In contrast, significant age-corrected correlations emerged between the Composite Vineland and total Profile scores, r (109) =-0.36, p <.001. Follow-up analyses revealed that that the Repetitive Questioning, Speech; Aggressive Behavior; and Hoarding Profile factors were negatively associated with the Vineland’s Daily Living Skills Domain; r’s (109) = − 0.40, − 0.37, and − 0.33, respectively, p’s < 0.001; and Socialization Domain; r’s (109) = − 0.34, − 0.35, and − 0.32, respectively, p’s < 0.001.

Gender. T-tests revealed just one significant gender difference, in the Hoarding factor, *t* (865) = -3.51, p <.001, *d* = 0.71. Females (M = 2.11, SD = 0.72) were more apt to engaging in non-food hoarding than males (M = 1.90, SD = 0.70).

Genetic Subtypes. Given the small number of persons with Imprinting Center Defects (ID, *n* = 27), t-tests compared participants with paternal deletions versus those with mUPD or ID. Combining these two subtypes is well-justified as each involves the silencing of imprinted paternal contributions, either by inheriting two maternal chromosomes (mUPD) or a defect in the imprinting center of the paternally inherited chromosome 15 (ID). Relative to those with paternal deletions, the combined mUPD and ID group scored significantly higher in the Repetitive Questioning, Speech factor, *t* (759) = -2.67, p =.008, *d* = 0.56, and the Depressed, Anxious factor, *t* (759) = -3.98, p <.001, *d* = 0.41. Participants with deletions, however, scored higher than their counterparts on Skin-Picking, *t* (759) = 3.37, p <.001, *d* = 0.68.

As the mUPD and ID subtypes are known to confer an increased risk of more severe psychopathology, t-tests were also conducted with the Red Flag items. Compared to those with deletions, the combined mUPD and ID group was more apt to run away, *t* (759) = -3.0, p =.003, *d* = 0.41, engage in rectal picking, *t* (759) = -2.28, p =.004, *d* = 0.58, exhibit self-harming behaviors, *t* (759) = -2.56, p =.01, *d* = 0.52, and to see or talk to people who are not present, *t* (759) = -3.61, p <.001, *d* = 0.67.

Age. Age was correlated with just one Profile factor, Hoarding (r =.30, p <.001). We further explored this finding by dividing participants into four developmentally appropriate age groups: children (5 to 12 years, *n* = 276), adolescents (13 to 19 years, *n* = 282), young adults (20–29 years, *n* = 184) and adults aged 30 or more years (*n* = 131). The ANOVA was significant, F (3,868) = 17.96, p <.001. The $\eta 2$ of 0.60 indicates a medium effect size [51]. Bonferroni post-hocs revealed that children had significantly lower scores (M = 1.78, SD = 0.66) than adolescents (M = 2.00, SD = 0.74), young adults (M = 2.19, SD = 0.67) and older adults (M = 2.27, SD = 0.70); both groups of adults also scored higher than adolescents.

BMI, Parental SES, Region. No significant correlations emerged between Profile factors and participant’s BMI, even when controlling for age. Similarly, no substantial effects were found for parental income, education, or regions where participants resided.

### Predictors of anxiety

The first regression model, with the CBCL Depressed/Anxious subdomain as the dependent variable, was significant, F (5,103) = 10.88, p <.001, adjusted *R*^*2*^ = 0.36. Two significant predictors were found, the Profile’s Depressed/Anxious factor (β = 0.315, p =.005), and the Distorted Negative Thinking factor (β = 0.230, p =.02).

The logistic regression model, with GAD as the dependent variable, was also significant, *X*^*2*^ (4) = 14.42, p =.006. The Nagelkerke R^2^ was 19.0%, and the model correctly classified 70.3% of participants. Two Profile factors were significant predictors of GAD. A one-unit increase on the Depressed, Anxious factor conferred a 4.30 increased likelihood of having GAD (odds ratio 95%CI = 1.24–15.50). The Repetitive Questioning, Speech factor was associated with a 3.04 increased likelihood of being diagnosed with GAD (Odds Ration 95% CI = 1.03–8.93).

## Discussion

PWS features a complex, multi-faceted behavioral phenotype. Normed on the general population or other developmental disabilities, existent measures do not readily capture the constellation of problems that are distinctive to PWS. This study fills the pressing need for a PWS-specific behavioral assessment that extends beyond measures of the syndrome’s characteristic hyperphagia.

In establishing the validity of this new instrument, our team first generated items based on our extensive work in PWS, and then obtained feedback about the items from multiple stakeholders—families, clinicians, and specialists in PWS. In a multi-phase process, items were revised or dropped, then pilot-tested, and additional feedback was obtained on pilot results from parents and a team of international experts in PWS. Based on their collective feedback, the Profile was again revised and completed by parents in a large-scale study. This iterative process, responsive to input from families and specialists, helped ensure the Profile’s construct and content validity.

Other psychometric properties of the Profile were also robust. Final factor analyses yielded eight conceptually meaningful domains of problematic behaviors and emotions in PWS, accounting for 60.5% of test variance. Factors were internally consistent, with strong alphas, and each item contributed meaningfully to their respective factors. Test re-test reliability showed some individual variation over time. Such findings bode well for the Profile to characterize natural variations in the phenotypic expression of PWS across development, and to gauge response to treatment. That said, however, ICCs were all moderate to good. And at the individual level, most participants had stable scores over time, with comparable percentages of participants who either improved or worsened over the 6 months.

Convergent validity of the Profile, assessed in pilot participants, revealed that Profile factors aligned very well with similar constructs on the CBCL or RBS-R. Beyond these logical relationships, other correlations are best understood in the context of the whole person with PWS. People with PWS, for example, often exhibit aggressive behaviors when environmental demands conflict with their rigid stances, compulsivity, or repetitive questioning. As well, it makes clinical sense that Profile factors tapping aggressivity, rigidity and negative affect would be associated with Social Problems on the CBCL.

Although IQ was not associated with Profile factors, higher total Profile scores were negatively associated with the Vineland Adaptive Behavior composite scores. In particular, the Aggressive Behaviors; Repetitive Questioning, Speech; and Hoarding factors were negatively associated with the Vineland’s Socialization and Daily Living Skills domains. As in other developmental disorders, these findings highlight the detrimental association between maladaptive behaviors and the performance of skills required for personal or social self-sufficiency [20, 54].

Genetic subtype differences in Profile factors and Red Flag items are consistent with previous work showing increased vulnerability to psychosis in people with mUPD, with or without a depressive component [55]. Relative to participants with paternal deletions, those with mUPD or Imprinting Deficits had higher scores on the Depressed, Anxious and Repetitive Questioning, Speech factors. They also had elevated scores on several Red Flag items that tapped symptoms of psychosis, and impulsive, risky behaviors. And, consistent with previous studies, participants with deletions were more prone to skin-pick than their counterparts [56].

Importantly, however, the magnitude of these genetic subtype differences was not large. Effect sizes clustered in the medium range, suggesting an overlap in the distribution of these problems across genetic subtypes. Thus, while those with mUPD or ID may be a higher risk for these problems, they are most certainly found in others with PWS.

Significant, medium-sized differences in age and gender were found in just one Profile factor, Hoarding. Elevated hoarding of non-food items has been reported in studies of compulsivity in PWS [57–60] but remains vastly understudied [61]. Hoarding behaviors were present in children and adolescents, but they were highest among younger and older adults, a trend also seen in people diagnosed with Hoarding Disorder [62]. Females with PWS were more apt to hoard non-food items than males. This gender difference, however, needs further study as it contradicts with the lack of gender biases for hoarding behaviors or Hoarding Disorder in the general population [62].

Regression analyses addressed the lingering question of how anxiety is manifest in PWS. Not surprisingly, the Profile’s Depressed, Anxious factor was a strong predictor of the CBCL’s Depressed/Anxious subdomain; the Distorted Negative Thinking factor was significant but had less predictive value. The logistic regression analysis further revealed that increased scores on Profile’s Depressed, Anxious factor conferred a 4.30-fold increased likelihood of having been diagnosed with Generalized Anxiety Disorder. Higher scores on the Repetitive Questioning, Speech factor conferred a 3.04-fold increased likelihood of this diagnosis.

It would be premature, however, to conclude that anxiety in PWS is only tapped by these two Profile factors, and that their compulsivity or rigidity do not also reflect anxiousness. People with PWS generally like performing their compulsive behaviors and typically become worried or upset when others interrupt or stop them. Distress and outbursts also typically ensue when their rigidity or needs for sameness are thwarted by a schedule change or unplanned circumstances. Similarly, anxiety in fragile X syndrome and ASD is characterized by increased compulsivity, repetitive speech, irritability and aggressivity [63, 64]. Such findings are consistent with the behavioral equivalents of anxiety for persons with intellectual disabilities as delineated in the DSM-ID-2 [23]. Although anxiety in PWS may thus be expressed differently from the general population, parents, clinicians, and individuals with PWS endorse anxiety as a salient feature of this syndrome [9, 24, 55, 65].

Several study limitations deserve mention. First, it was not feasible to include convergent validity measures in the PWS Global Registry. As such, convergent validity analyses were only conducted with pilot participants. We offset this limitation by ensuring participants did not differ across the two recruitment sources in demographics or Profile scores. Second, test-retest reliability analyses are typically conducted across shorter time frames than this study’s 6-month test-retest interval. Nevertheless, ICCs were good, and at the individual level, most participants showed stable scores.

Additionally, we did not assess the divergent validity of the Profile, primarily because our goal was to portray the constellation of problematic behaviors that are particular to PWS. Future research may reveal areas of both continuity and discontinuity in Profile factors relative to other neurodevelopmental disorders. Finally, the lack of Profile differences related to BMI, IQ, parental SES, or region, and the few differences related to age and gender, speak to the broad applicability of Profile. Even so, future Profile studies are needed with more diverse samples to verify these results.

Despite these limitations, the PWS Profile emerged as a valid, psychometrically-sound instrument that holds considerable promise for future research. Although people with PWS are genetically predisposed to exhibit certain behaviors, the Profile can advance studies on environmental factors that also powerfully shape their behaviors, including life experiences, learning, interventions, family background and genetics, and development across the life span. As well, the Phase 3 pilot version of the Profile was used as an endpoint in a previous clinical trial, showing a robust response to treatment [34]. The Profile may thus extend the reach of future clinical trials by capturing the impact of pharmaceutical agents not only on measures of hyperphagia, but also on the range of emotional and behavioral problems that characterize PWS.

### Electronic supplementary material

Below is the link to the electronic supplementary material.

Supplementary Material 1

Supplementary Material 2

## Data Availability

The data that support the findings of this study are available from the Foundation for Prader-Willi Research (FPWR), but restrictions apply regarding the availability of these data, as they were gathered with permission and additional internal approval from FPWR. Data are available from the authors upon reasonable request and with permission and additional approval from FPWR.
