# Supplementary material for: The Prader-Willi syndrome Profile: validation of a new measure of behavioral and emotional problems in Prader-Willi syndrome
Source: Orphanet J Rare Dis. 2024 Feb 23;19:83. doi: 10.1186/s13023-024-03045-9 (PMC10885615; doi:10.1186/s13023-024-03045-9)
Supplement: Supplementary file 1 — Supplementary Material 1 [file 13023_2024_3045_MOESM1_ESM.docx]

**Additional File 1**

The following 18 Items were omitted after the Phase 3 pilot study due to either low frequency of endorsement or item redundancy.

**Low Frequency Items:**

Collects items in a certain order (e.g., must have 1st in a series before getting 2nd, must get all a series at once) or until the set is complete.

Hand wringing or flapping when excited or nervous (e.g., moves hands up/down or rubs them together).

Engages in self-soothing activities (e.g., shreds paper, rubs hand on pants, twirls string, etc.).

Generally, shows little facial response to positive things and if so, it disappears quickly.

Holds grudges, doesn’t forget others past mistakes.

Difficulty maintaining appropriate space boundaries with others (e.g., stands too close or too far, gets too close to a person’s face).

Laughs at others when they have an accident or mishap (e.g., someone drops something, bumps their knee, gets a paper cut).

Difficulty matching his/her emotions or actions to situations (e.g., smiles or is unsympathetic in sad situations, laughs at the wrong times).

Distrustful of others (judges others quickly or harshly).

Decides right away if someone doesn’t like them.

Has difficulty recognizing people he/she knows (e.g., parents, teachers).

Disrobes when faced with intense emotional state.

Creates situations to get into the hospital or attention from the police or health professionals.

Reports that people are out to get him/her or are talking behind his/her back.

**Redundant Items:**

Finds other people frustrating or annoying (may be irritable or cranky with them).

Withdraws from or avoids activities or people that he/she previously enjoyed.

Insists that certain people say things in certain ways (may seem scripted or repetitive).

Looks to pick a fight, provokes others.
