# Supplementary material for: The Prader-Willi syndrome Profile: validation of a new measure of behavioral and emotional problems in Prader-Willi syndrome
Source: Orphanet J Rare Dis. 2024 Feb 23;19:83. doi: 10.1186/s13023-024-03045-9 (PMC10885615; doi:10.1186/s13023-024-03045-9)
Supplement: Supplementary file 2 — Supplementary Material 2 [file 13023_2024_3045_MOESM2_ESM.docx]

**Additional File 2**

**Three Items Omitted due to Redundancy in Final Factor Analyses**.

Prefers to spend time alone.

Needs to be in control, is bossy, tells others how things must be done.

Asks intrusive or inappropriate questions.

**Three items Omitted due to Poor Loading in Final Factor Analyses.**

Inflated sense of self (e.g., unrealistic expectations of their own limitations)

Creates situations to avoid demands (e.g., causes problems at school to be sent home, spends time in the bathroom to avoid class, refuses to go to work),

Talks with dolls, belongings, stuffed animals in imaginary scenarios (e.g., plays teacher to pretend students)
